# Carbon Source Influences Antioxidant, Antiglycemic, and Antilipidemic Activities of *Haloferax mediterranei* Carotenoid Extracts

**DOI:** 10.3390/md20110659

**Published:** 2022-10-24

**Authors:** Micaela Giani, Luigia Gervasi, Monica Rosa Loizzo, Rosa María Martínez-Espinosa

**Affiliations:** 1 Biochemistry and Molecular Biology Division, Agrochemistry and Biochemistry Department, Faculty of Sciences, University of Alicante, Ap. 99, E-03080 Alicante, Spain; 2 Multidisciplinary Institute for Environmental Studies “Ramón Margalef”, University of Alicante, Ap. 99, E-03080 Alicante, Spain; 3 Department of Pharmacy, Health Science and Nutrition, University of Calabria, I-87036 Arcavacata Rende, Italy

**Keywords:** *Haloferax mediterranei*, bacterioruberin, haloarchaea, hyperglycemia, obesity, carotenoids

## Abstract

Haloarchaeal carotenoids have attracted attention lately due to their potential antioxidant activity. This work studies the effect of different concentrations of carbon sources on cell growth and carotenoid production. Carotenoid extract composition was characterized by HPLC-MS. Antioxidant activity of carotenoid extracts obtained from cell cultures grown under different nutritional conditions was determined by 2,2′-azino-bis (3-ethylbenzothiazoline-6-sulfonic acid) (ABTS) and 1,1-diphenyl-2-picrylhydrazyl (DPPH), Ferric Reducing Ability Power (FRAP) and β-carotene bleaching assays. The ability of these carotenoid extracts to inhibit α-glucosidase, α-amylase, and lipase enzymes was also assessed to determine if they could be used to reduce blood glucose and lipid absorption. The maximum production of carotenoids (92.2 µg/mL) was observed combining 12.5% inorganic salts and 2.5% of glucose/starch. Antioxidant, hypoglycemic, and antilipidemic studies showed that higher carbon availability in the culture media leads to changes in the extract composition, resulting in more active haloarchaeal carotenoid extracts. Carotenoid extracts obtained from high-carbon-availability cell cultures presented higher proportions of all-*trans*-bacterioruberin, 5-*cis*-bacterioruberin, and a double isomeric bacterioruberin, whereas the presence 9-*cis*-bacterioruberin and 13-*cis*-bacterioruberin decreased. The production of haloarchaeal carotenoids can be successfully optimized by changing nutritional conditions. Furthermore, carotenoid composition can be altered by modifying carbon source concentration. These natural compounds are very promising in food and nutraceutical industries.

## 1. Introduction

Carotenoids are natural compounds of high-biotechnological and biomedical interest. They are well known for their beneficial effects on human health, given their antioxidant properties [1,2,3,4]. 

Carotenoids can be classified depending on the length of their carbon chain in C_30_, C_40_, and C_50_, meaning 30, 40, and 50 carbon units conforming their structure, respectively [5]. C_50_ carotenoids are mainly synthesized by halophilic archaea (also termed haloarchaea), which are extremophilic microorganisms that belong to the Archaea domain [6,7]. Since these microorganisms usually inhabit hypersaline environments, they require high salt concentrations for their survival in culture media [8,9]. 

Haloarchaea produce C_50_ carotenoids as a defense mechanism against osmotic stress and the radiation naturally present in their habitats [10]. Bacterioruberin (BR) and its derivatives, monoanhydrobacterioruberin (MABR) and bisanhydrobacterioruberin (BABR) have been reported as the main components of haloarchaeal carotenoid extracts [11,12]. BR is formed by a primary isoprenoid chain which includes thirteen conjugated double bonds and four hydroxyl groups in the terminal ends [13]. These characteristics a priori provide BR with higher antioxidant properties than other C_40_ and C_30_ carotenoids, such as β-carotene and staphyloxanthin [14,15,16]. In consequence, BR could be of great interest to the food and nutraceutical industries due to its potential in different biomedical areas: cancer, antiviral [17], and sperm motility treatments [18]. Recent studies have brought some light regarding the antioxidant properties of haloarchaeal carotenoid extracts [18,19,20]. However, little is known about the effect of nutritional conditions on the antioxidant properties and potential biological activities of these extracts. 

Microbial production of carotenoids has gained attention in the last years due to its potential as a more economic and respectful to the environment approach [16,21,22,23]. In particular, haloarchaeal cell cultures have a lower risk of contamination thanks to the high concentration of salt, thus sterilization not being required. Furthermore, cell lysis can be easily carried out by exposure to a hypotonic solution [24]. These particularities translate into a lower cost of production [25]. Among haloarchaea, *Haloferax mediterranei* stands out for its versatility in consuming carbon sources and the extensive available bibliography regarding its metabolism [26,27,28,29]. *Hfx. mediterranei* has proved to be useful in the production of bioplastics like polyhydroxyalkanoates (PHA) [30,31,32,33] and is considered a model microorganism in a variety of fields, including the nitrogen cycle [28,34].

Lately there has been an expanding interest in the production and industrial application of haloarchaeal C_50_ carotenoids [6,16,35,36]. However, there is still much work to be done in the optimization of the cell culture media composition to enhance the production to its maximum while reducing costs. A low concentration of salt has been demonstrated to be key in the induction of BR synthesis in *Hfx. mediterranei* [29,37]. Nevertheless, little is known about the relevance of the use of different carbon sources on the growth and the synthesis of haloarchaeal carotenoids. 

In this study, haloarchaeal carotenoids were tested as potential inhibitors of α-glucosidase, α-amylase, and pancreatic lipase, since diabetes and obesity prevalence keeps increasing each year and has already reached epidemic proportions [38,39,40]. There is a growing concern about the consequences of the current situation since both pathologies are risk factors for the development of cardiovascular diseases [41,42]. Among antidiabetic drugs, α-glucosidase and α-amylase inhibitors are used for the treatment of diabetes mellitus type 2 patients or of those patients at risk of hypoglycemia or acidosis with the aim of delaying the development of diabetes [43,44,45]. Inhibitors of pancreatic lipase are particularly useful to decrease the absorption of fats in obese patients, thus helping in weight loss [46,47].

In this work, we have tested the effect of different carbon sources on *Hfx. mediterranei* growth, carotenoid production, and the properties of the carotenoid extracts obtained under the different nutritional conditions. The in vitro antioxidant properties as well as carbohydrate-hydrolyzing enzymes and lipase inhibitory activities were assessed to explore biomedical applications of haloarchaeal carotenoids. 

## 2. Results and Discussion 

### 2.1. Effect of Different Carbon Sources on Growth 

**Figure 1:**
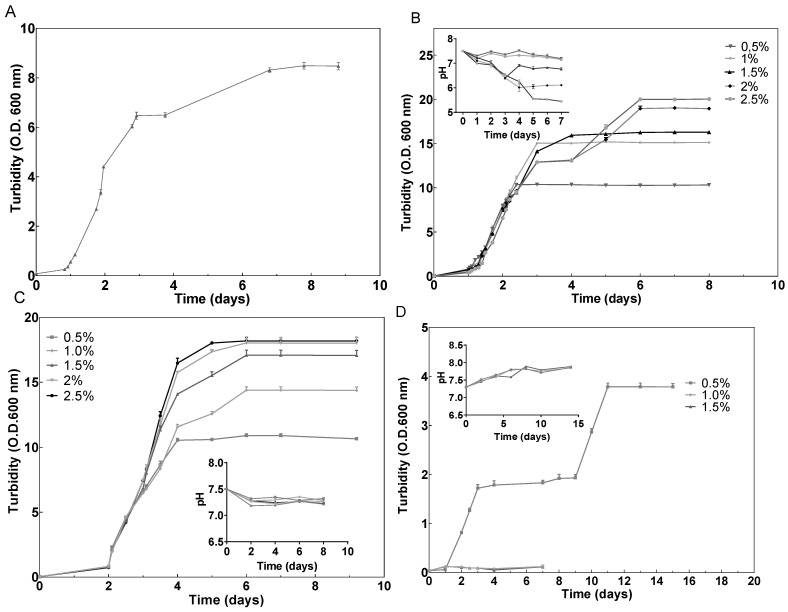
Effect of (**A**) no additional carbon source (control), (**B**) glucose (0.5–2.5% (*w*/*v*)), (**C**) starch (0.5–2.5% (*w*/*v*)), and (**D**) oxalacetate (0.5–1.5% (*w*/*v*)) on time course of growth (OD600 nm) of *Hfx. mediterranei*.

**Figure 2:**
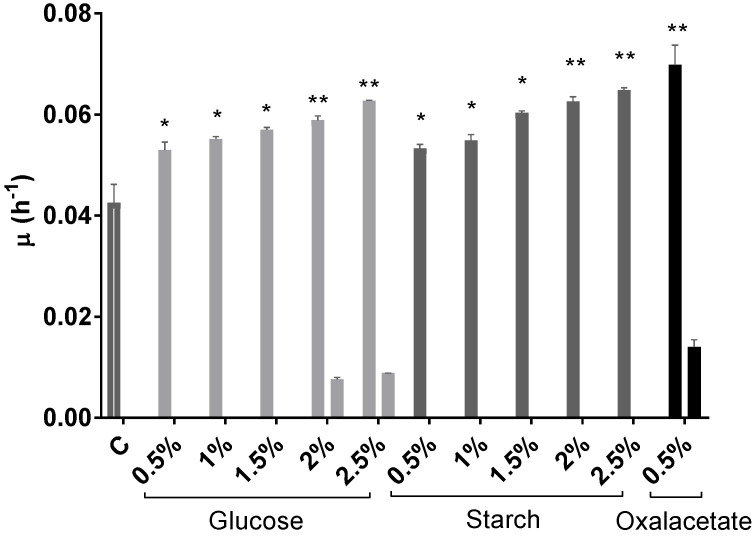
Effect of glucose (0.5–2.5% (*w*/*v*)), starch (0.5–2.5% (*w*/*v*)), and oxalacetate (0.5% (*w*/*v*)) on growth-specific velocity in *Hfx. mediterranei* cell cultures. Control was a cell culture medium with no additional carbon source. Each experimental sample was compared to the control to evaluate statistical significance. * *p* < 0.05, ** *p* < 0.01.

*Hfx. mediterranei* grew very efficiently in the presence of glucose; however, the highest concentrations (2% and 2.5% (*w*/*v*)) produced a dramatic drop in pH, probably due to the products of glucose metabolism via the Entner–Doudoroff pathway (Figure 1B) [48]. Elevated concentrations of glucose have been proven efficient in inducing cell growth also in other haloarchaeal species, such as *Halorubrum* sp. M5 [49]. Regarding starch, the cells grew successfully, and in this case without affecting the pH value of the cell culture (Figure 1C). These results agree with previous results that demonstrated that starch could be metabolized by *Hfx. mediterranei* [50]. Furthermore, as it can be observed in Figure 1, growth was concentration-dependent, leading to OD_600_ values significantly higher than those obtained in the cell culture without an additional carbon source (Figure 1A). The same tendency was confirmed by the obtention of the growth-specific velocities for each cell culture (Figure 2). With respect to oxalacetate, this intermediate of the tricarboxylic acid (TCA) cycle was tested in three different concentrations: 0.5%, 1%, and 1.5%, and the cells were able to grow only in the presence of 0.5%. However, the slow and diauxic growth observed indicates that cells had difficulties in metabolizing this compound (Figure 1D). There was a positive correlation between the concentration of the carbon source in the culture media and the specific growth velocity, even in the cases of 2% and 2.5% of glucose where diauxic growth was observed (Figure 2).

### 2.2. Effect of Different Carbon Sources on BR Production

Glucose was remarkably effective in inducing carotenoid production, coinciding with previous results in other species [49,51].

**Figure 3:**
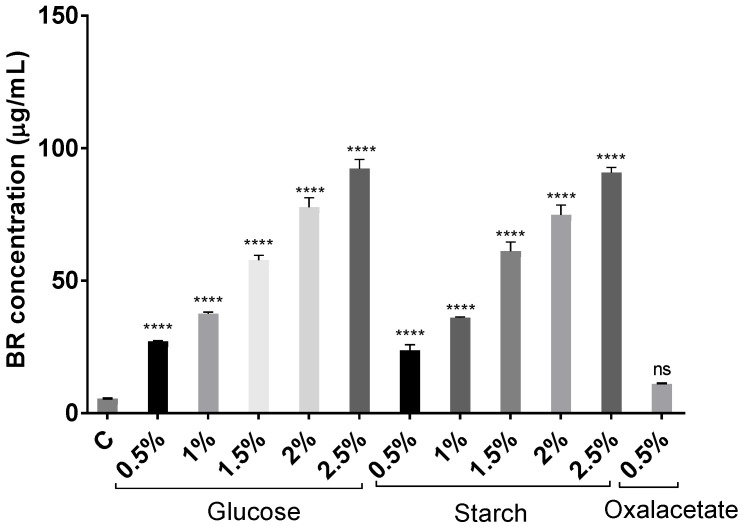
Effect of glucose (0.5–2.5% (*w*/*v*)), starch (0.5–2.5% (*w*/*v*)), and oxalacetate (0.5% (*w*/*v*)) on BR production in *Hfx. mediterranei* carotenoid extracts. Each experimental sample was compared to the control to evaluate statistical significance. ns: not significant, **** *p* < 0.0001.

**Figure 4:**
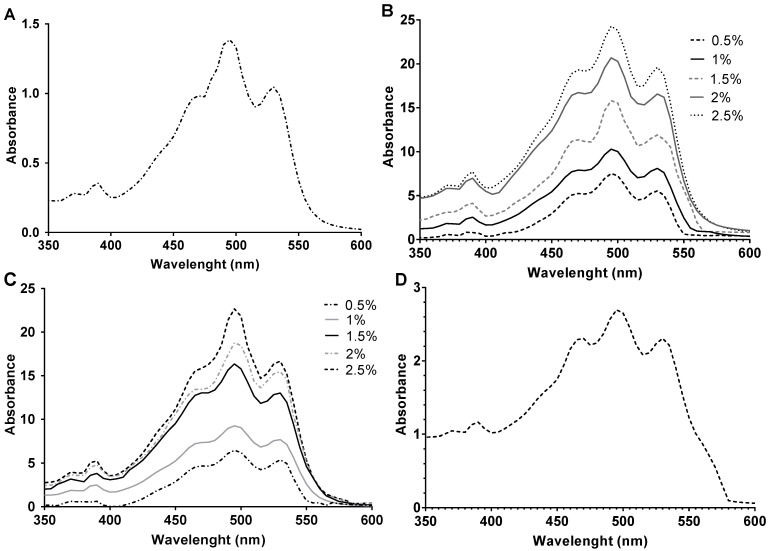
UV-Vis Spectra (350–600 nm) of carotenoid extracts from *Hfx. mediterranei* growth with (**A**) no additional carbon source, (**B**) glucose (0.5–2.5% (*w*/*v*)), (**C**) starch (0.5–2.5% (*w*/*v*)), and (**D**) oxalacetate (0.5% (*w*/*v*)).

The increment of carbon source concentration not only favors growth but also induces carotenoid synthesis since carbon is the main component of the structure of carotenoid molecules. Figure 3 and Figure 4 show how the BR concentration was higher in those carotenoid extracts obtained from cell cultures where there was more carbon available. The highest concentration was obtained from the cell culture with 2.5% of glucose. However, the results obtained with starch were very similar. Therefore, both carbon sources were greatly effective in inducing carotenogenesis. In addition, all extracts obtained from glucose and starch cell cultures had significantly higher BR concentrations than the extracts obtained from the cell culture with no additional carbon source (control) did. Interestingly, the extract obtained from *Hfx. mediterranei* grown in the presence of oxalacetate led to high absorbance at 388 nm, which might indicate abundance of *cis*-BR-isomers [19,52]. Meléndez-Martínez and collaborators described that when the *cis*-double bond is closer to the center of the BR molecule, the absorbance at 388 nm is more intense [53].

Despite the differences in methods and expression of units, the combination of low salt content and high carbon availability has led to optimal production of C_50_ carotenoids, surpassing most results in the literature [19,24,25,49,54,55,56] with the exception of [50], in which a maximum of 556 mg/L of total carotenoids was achieved in the presence of a high salt concentration. In order to compare the methods and results reported in Chen et al., 2014 with those summarized here, their maximum production of carotenoids was reached at a constant conductivity of 25 S/m, which represents approximately a total salt concentration of 144 g/L, which is very similar to the approximately 141 g/L of salt (12.5% SW) used in our experiments. The quantification of pigments was carried out by measuring the absorbance at 495 nm, which coincides with the selected wavelength for our assays (494 nm), which is the maximum of absorbance of BR. However, there is no information about the formula used to calculate the concentration from the absorbance value. Therefore, it is difficult to conclude why we observed such differences in BR production, but it might be due to the maintenance of the conductivity constant and/or variations in the formula used to calculate the concentration of BR. 

The combination of higher concentrations of the carbon source with growth parameters, such as salt concentration, are key for the optimization of the production of haloarchaeal carotenoids. Recent results also indicate that nutrient deprivation could enhance BR production [29]. However, our results support that carbon excess in combination with low salinity (12.5%) and low temperature (36.5 °C) conditions offer *Hfx. mediterranei* the necessary resources to synthesize more BR to overcome osmotic stress. 

### 2.3. Antioxidant Activity

Haloarchaeal carotenoid extracts from the 2.5% glucose cell culture showed the highest activity in DPPH and ABTS tests, with IC_50_ of 32.4 and 0.03 µg/mL, respectively (Table 1). In fact, all haloarchaeal carotenoid extracts were more effective than ascorbic acid in scavenging the ABTS radical cation. The obtained IC_50_ values showed also that the antioxidant potential of these carotenoid extracts was higher than those reported for other haloarchaeal species. For example, concentrations higher than 16 µg/mL would be required to scavenge at least 50% of ABTS radicals with carotenoid extracts from *Halococcus morrhuae*, *Halobacterium salinarum*, and *Thermus filiformis* [52]. Other IC_50_ values reported for this assay are: 0.8 µg/mL in *Haloterrigena* sp. strain SGH1 [19], 3.89 µg/mL in *Haloarcula hispanica* HM1 [57], a range of 4.23–34.7 µg/mL in six different haloarchaeal strains (Te Se-85, Te Se-86, ALT-23, TeSe-41, TeSe-51 and TeSe-89) [58], and 3–6 µg/mL in *Halorubrum* sp. BS2 [59]. Interestingly, this same study on *Halorubrum* sp. BS1 presented values between 6 and 9 µg/mL for the 50% inhibition of the DPPH radical, which are concentrations lower than those detected in *Hfx. mediterranei* extracts. These differences between assays might be attributed to the composition of each haloarchaeal carotenoid extract which might interact differently with the used radicals. It is also worth mentioning that the antioxidant effects of ascorbic acid vary between studies, which might be due to variations in the methodologies applied. Regarding other extracts, astaxanthin has been reported to inhibit 50% DPPH at a concentration around 400 µM [60], which is much higher than the values obtained for *Hfx. mediterranei* extracts. *Dunaliella salina* is a well-known producer of carotenoids, particularly β-carotene, whose extracts’ EC_50_ value is 8360 µg/mL [61]. Other studies have reported an inhibition of DPPH around 50% at a concentration of 250 µg/mL of a *D. salina* carotenoid extract [62]. Thus, *Hfx. mediterranei* extracts are between 8 and 260 times more effective in scavenging DPPH radicals than *D. salina* extracts. Other commonly commercialized microorganism as an antioxidant supplement is *Spirulina platensis*, whose EC_50_ values have been reported to be 449 µg/mL and 5852 µg/mL for the DPPH and ABTS tests, respectively [63]. Therefore, *Hfx. mediterranei* is a promising source of antioxidant compounds. 

When compared to the standard control (complex media without an additional carbon source), all extracts presented lower IC_50_ values, indicating that the addition of carbon sources to the media not only increased the production of carotenoids but changes the composition of the extract, thus leading to differences in the exerted antioxidant activity. There is a trend towards an inverse correlation between the concentration of BR present in the extract and the IC_50_ from all assays. In the FRAP test, the most active haloarchaeal carotenoid extracts were those obtained from cell cultures with concentrations of glucose of 1.5%, 2%, and 2.5% (30.7, 38.7, and 39.6 µg/mL, respectively), reducing higher concentrations of Fe^3+^ than with the positive control BHT. These results are also supported by a Pearson’s correlation coefficient of 0.81. Regarding the β-carotene bleaching assay, the highest antioxidant activity was observed in extracts obtained from cell cultures containing 2.5% of glucose or starch (0.15 µg/mL in both cases after 30 min of incubation; and 0.47 and 1.1 µg/mL after 60 min, respectively). Pearson’s correlation coefficients, which evaluated the correlation between the concentration of BR and the antioxidant activity exerted by haloarchaeal carotenoid extracts (IC_50_) were 0.76, 0.71, 0.74, and 0.84 for the DPPH assay, ABTS, and β-carotene bleaching assay at 30 and 60 min, respectively.

**Figure 5:**
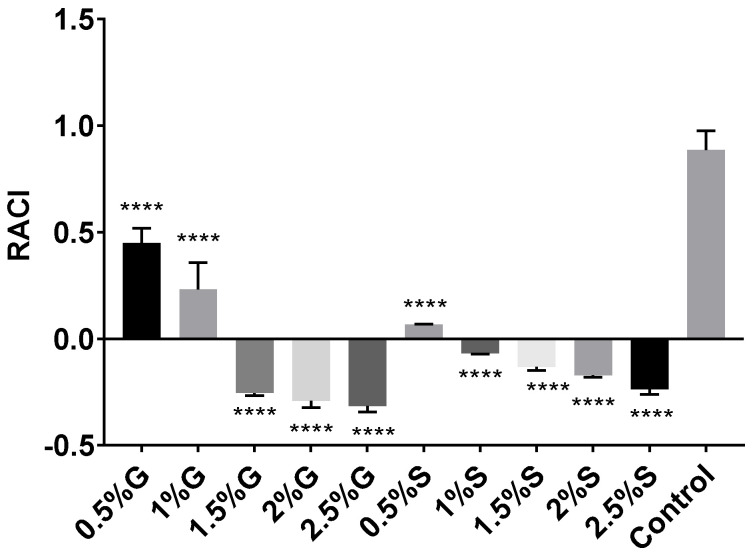
Relative antioxidant capacity index (RACI) of haloarchaeal carotenoid extracts from *Hfx. mediterranei* growth in the presence of glucose, starch, or neither. RACI values were developed from data obtained from the antioxidant methods applied. Each experimental sample was compared to the control to evaluate statistical significance. **** *p* < 0.0001.

Relative antioxidant capacity index (RACI) can be used as a reference for determining antioxidant capacity by combining the results of different techniques [64]. Even though RACI is a relative index and cannot provide specific data about antioxidant properties, it is useful for obtaining an integrated and combined vision of the antioxidant capacity of an extract. Therefore, data from DPPH, ABTS, FRAP, and β-carotene bleaching assays were used to determine the RACI value for each carotenoid extract obtained from *Hfx. mediterranei* cells grown under different nutritional conditions previously commented (See Section 2.1 and Section 3.1.1). The results are shown in Figure 5. RACI calculations represent the average of the standard scores obtained from the IC_50_ values for the different methods used. Thus, the lower the RACI value, the higher the antioxidant activity (Figure 5). The results supported the results presented in previous sections. The extracts obtained from high-carbon-availability cell cultures presented higher antioxidant capacity (RACI < 0) than those obtained from cells grown in the presence of low concentrations of glucose or starch (RACI > 0).

### 2.4. Antiglycemic and Antilipidemic Activity 

A correlation between oxidative stress, obesity, and type 2 diabetes has been repeatedly reported [65,66]. α-glucosidase enzyme has been reported as a relevant therapeutic target for the management of diabetes mellitus due to its confined effect in the intestine [67]. Many plant extracts exert this type of activity [68,69,70,71,72]. However, this potential biomedical strategy has not yet been evaluated in carotenoid extracts and even less in haloarchaeal carotenoid extracts. Consequently, the ability to inhibit α-glucosidase, α-amylase, and lipase was evaluated in *Hfx. mediterranei* carotenoid extracts (Table 2). All extracts obtained from 1% to 2.5% glucose or starch cell cultures were significantly more effective than the extract of a commercial drug, acarbose (35.6 ± 0.91 and 50.2 ± 1.3 µg/mL for α-glucosidase and α-amylase, respectively). The highest activity was observed in the extract from the 2.5% glucose cell culture, with an IC_50_ value of 3.2 µg/mL for α-glucosidase and 1.3 µg/mL for α-amylase. The IC_50_ value for α-glucosidase was lower than those reported for many plant species, such as *Ipomoea batatas* (4.5–181.9 µM) [68], *Morus atropurpurea* (13.2–365.4 µM) [70], *Camellia sinensis* (299 µg/mL) [73], and phenolic extracts from *Spirulina* sp. powder (1670 µM) [74]. *Hfx. mediterranei* extracts were also more effective in inhibiting α-amylase than other natural compounds were, like phycocyanin from *Spirulina platensis* (IC_50_ of 150–200 µg/mL) [75], polyphenol and fucoxanthin-rich extract from *Sargassum hemiphyllum (*(IC_50_ of 350 µg/mL) [76], and *Chaetomorpha aerea* extract (IC_50_ of 408.9 μg/mL) [77].

Pancreatic lipase is an enzyme that plays a key role in the digestion of lipids. It is responsible for the breakdown of triglycerides in the gastrointestinal tract so that they can be absorbed in the form of fatty acids [78]. When inhibited, a hypolipidemic effect appears, since fewer fats are absorbed [79]. The highest inhibitory activity has been detected in those extracts from 2.5% glucose or starch cell cultures, with IC_50_ values of 5.3 µg/mL, which was significantly lower than those observed in the internal control (78.4 µg/mL) and those from the commercial drug Orlistat (37.5 µg/mL), respectively. Pearson’s correlation coefficient demonstrated a clear correlation (*r* = 0.9) between the concentration of BR in haloarchaeal extracts and their inhibitory lipase activity. Lipase inhibition by *Hfx. mediterranei* carotenoid extracts was stronger than by others previously reported: flavonoids from *Litchi chinensis* that reach a maximum of inhibition of 44.69% with a concentration of 7000 µg/mL [80], Daisaikoto extract (IC_50_ = 13,400 µg/mL) [81], and *Crocus cancellatus* subsp. *damascenus* extract (5000 µg/mL) [82].

### 2.5. Carotenoid Composition by HPLC-MS

**Figure 6:**
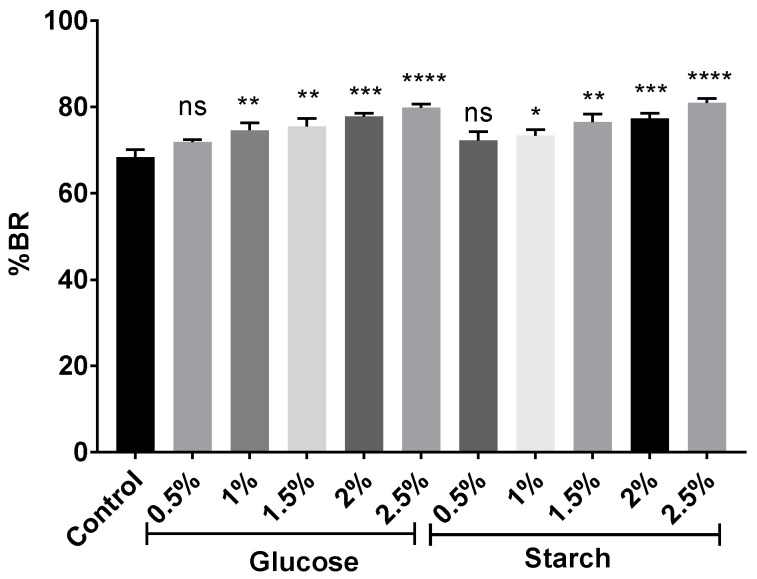
Percentage of total BR in relation to the total of carotenoids identified in each sample. Each experimental sample was compared to the control to evaluate statistical significance. ns: not significant, * *p* < 0.05, ** *p* < 0.01, *** *p* < 0.001, **** *p* < 0.0001.

The carotenoid extracts obtained from the cell cultures supplemented with 0.5–2.5% glucose or starch were analyzed by HPLC-MS to determine their composition. The *m*/*z* value (740.6) for BR was detected at several retention times along the elution, ranging from 3 to 8 min. These results indicated the presence of different BR isomers. In Figure 6, it can be observed how the total amount of bacterioruberins (without taking into consideration the different isomers) slightly increased in a positive correlation with the concentration of the carbon source in the culture media from where they were obtained. BR constituted between 68% and 81% of the identified carotenoids, coinciding with previous results that show this carotenoid as the major constituent of haloarchaeal carotenoid extracts [19,35,83,84,85].

There is very little information available regarding the identification of BR isomers. Flores and collaborators reported the detection of different BR isomers in *Haloterrigena* sp. strain SGH1, with retention times between 10.4 and 12.1 min [19]. Later, Lizama et al., 2021 analyzed the composition of carotenoid extracts obtained from six different species belonging to the genus *Haloarcula* and *Halorubrum*, which, instead, eluted between 4.45 and 6.31 min [58]. In addition, they identified for the first time double isomeric BR forms, with eluted at 7 and 8 min, respectively. In our carotenoid extracts, BR eluted from 3 to 8 min, which coincides closely with the results of [58]. Given the lack of knowledge and the differences in techniques, it is difficult to compare the results. However, what seems to be clear is that the different isomers elute in a particular order. We identified five different retention time zones in which BR was identified (Table 3), which agree with the isomers reported in the bibliography [19,58]. The detection of BR between 7 and 7.9 min indicates the presence of a double isomeric form [58]. 

**Figure 7:**
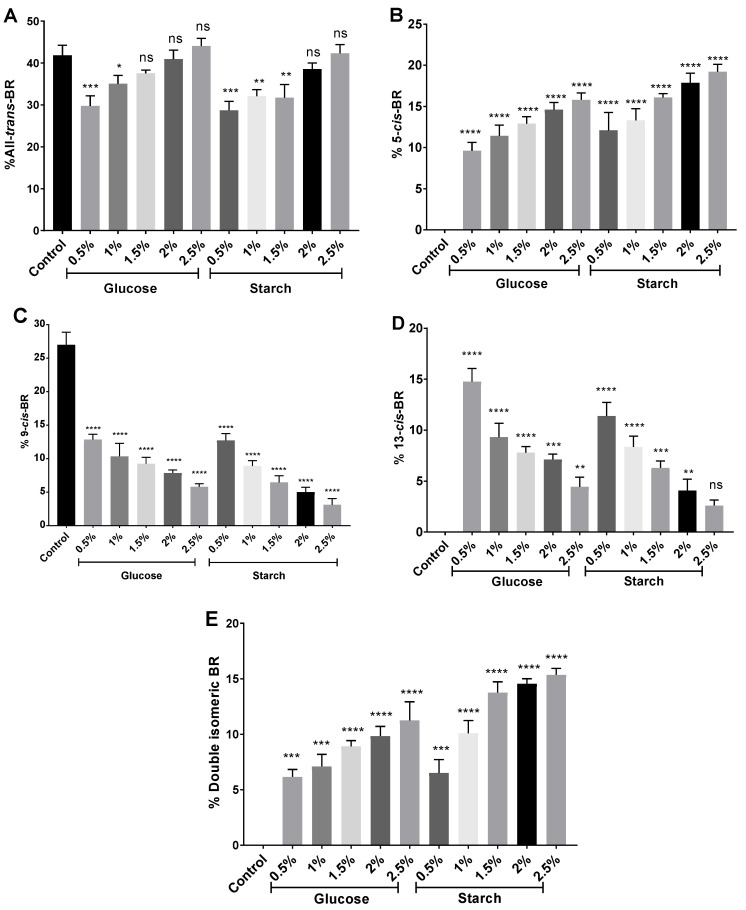
Percentage of (**A**) all-*trans*-BR, (**B**) 5-*cis*-BR, (**C**) 9-*cis*-BR, (**D**) 13-*cis*-BR, and *(***E**) double isomeric-BR in relation to the total of carotenoids identified in each sample. Each experimental sample was compared to the control to evaluate statistical significance. ns: not significant, * *p* < 0.05, ** *p* < 0.01, *** *p* < 0.001, **** *p* < 0.0001.

Once the different isomers were identified by their retention times, the percentage that each of them represented over the total of carotenoids was calculated (See Section 3.5) (Figure 7). All-*trans*-BR was the most abundant BR isomer representing between 28% and 44% of the total carotenoid content, similar to what was previously described [19], and moderately less than reported by Mandelli and collaborators [52]. The internal control, an extract from a cell culture without a supplementary carbon source, presented only all-*trans*-BR and 9-*cis*-BR isomers; while the rest of the extracts, obtained from cells exposed to higher concentrations of carbon, were composed of a mix of all isomers (all-*trans*-BR, 5-*cis*-BR, 9-*cis*-BR, 13-*cis*-BR and at least a double isomeric BR form). 5-*cis*-BR, 9-*cis*-BR, and 13-*cis*-BR were found in percentages of 9.6–19.2%, 5.8–12.8%, and 2.6–14.8%, respectively, which is in agreement previous results [19]. Therefore, the nutritional conditions influence the BR isomers produced by *Hfx. mediterranei.* In fact, increasing concentrations of carbon led to higher percentages of all-*trans*-BR, 5-*cis*-BR, and the double isomeric BR form, whereas the presence of 9-*cis*-BR and 13-*cis*-BR was reduced. The observed tendency was comparable in extracts obtained from both glucose- and starch-supplemented cell cultures. Thus, it seems more related to the concentration than to the type of carbon source. A previous study on *Halococcus morrhuae* and *Halobacterium salinarium* concluded that 13-*cis*-BR was more abundant than 9-*cis*-BR and 5-*cis*-BR in their carotenoid extracts [52], which differs from our findings in *Hfx. mediterranei.*

**Figure 8:**
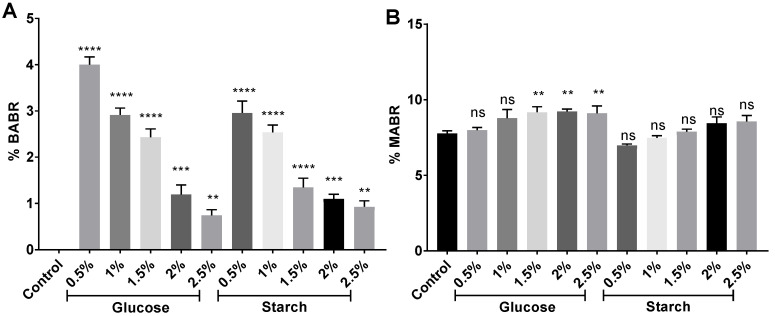
Percentage of (**A**) monanhydro-BR (MABR) and (**B**) bisanhydro-BR (BABR) in relation to the total of carotenoids identified in each sample. Each experimental sample was compared to the control to evaluate statistical significance. ns: not significant, ** *p* < 0.01, *** *p* < 0.001, **** *p* < 0.0001.

Monoanhydrobacterioruberin (MABR) and bisanhydrobacterioruberin (BABR) were detected in the presence of 7.8–9.1% and 0.7–4.0% (Figure 8), respectively, which agrees with other studies [83]. MABR abundance slightly increased in the extracts from high-carbon-availability cell cultures, whereas BABR clearly decreased. Trisanhydrobacterioruberin (TABR) was also detected in all extracts, as previously reported [86].

Xanthophylls were detected in all extracts except the control without an additional carbon source. Particularly, cantaxanthin percentage increased with carbon availability in the culture media up to 3.9% of total carotenoids. Astaxanthin and zeaxanthin were also identified in percentages of 0.6–1.7% and 0.1–1.5%, respectively. They showed an inverse correlation with the concentration of carbon in the culture media. Previous publications have also described the synthesis of xanthophylls in halophilic microorganisms [36,85,87].

Herein, we report for the first time the antioxidant profile of *Hfx. mediterranei* carotenoid extracts obtained under different nutritional conditions. The combination of low salinity and elevated carbon availability led to one of the highest concentrations of BR reported in the literature. These results have shown how carbon source concentration can modify the composition and, in consequence, the properties of *Haloferax mediterranei* carotenoid extracts, which is of great relevance in the optimization process of the production of these compounds for further biotechnological or biomedical application. Furthermore, haloarchaeal carotenoids have proven to be very successful in the inhibition of enzymes involved in the digestion of carbohydrates and lipids, which opens a new window for their application in biomedicine. These findings are key to improve haloarchaeal carotenoid production and their use as nutraceuticals.

## 3. Materials and Methods

### 3.1. Culture Growth Conditions

#### 3.1.1. Cultivation in the Presence of Different Concentrations of Carbon Sources 

*Hfx. mediterranei* strain R-4 (ATCC33500) was used for all experiments. Cells were grown in a complex medium containing 12.5% (*w*/*v*) of inorganic salts [29,88], 0.5% (*v*/*w*) yeast extract, and 0.5–2.5% (*w*/*v*) of a carbon source (glucose (BioChemica, CAS: 50-99-7), soluble starch (Merck, CAS:9005-84-9) or oxalacetic acid (Alfa Aesar CAS: 328-42-7)). pH was buffered using 30 mM Tris-HCl (pH 7.3). Growth conditions included 36.5 °C and shaking at 170 rpm (Infors HT Multitron Standard) based on the data reported by [24]. Cultures were incubated until three days had passed after the maximum of absorbance was reached (stationary phase of growth). Since BR is a secondary metabolite, the stationary phase is where the majority of BR is synthesized. Then, the cells were centrifuged at 7800 rpm for 30 min to remove the supernatant. The cells were washed twice with a 10% (*w*/*v*) inorganic salts solution. Growth-specific velocity (µ) Equation (1) and duplication time (Dt) Equation (2) were calculated for each growth condition as follows:

µ = ln (X − X_0_)/(t − t_0_), (1)

Dt = ln(2)/µ(2)

#### 3.1.2. Growth Determination

Growth was determined by measuring the turbidity of the culture at 600 nm using a UV-Vis spectrophotometer (Agilent, Santa Clara, CA, USA).

### 3.2. Carotenoid Extraction and BR Quantification

Cell pellets were suspended in pure acetone in a ratio of 1 mL of acetone per 10 mL of cell culture. Then, they were incubated at 4 °C overnight and then centrifuged (7800 rpm, 30 min) to obtain the carotenoid extract. BR concentration was calculated using the following expression [24]:

mg·L^−1^ = (OD_494_/2540) × 10^4^ (3)

BR extracts were stored at −20 °C in a solution. 

Absorption UV–visible spectra were obtained at room temperature on a Cary 60 UV-Vis spectrophotometer (Agilent, Santa Clara, CA, USA) using the scan mode, with a 300–800 nm absorbance range. Acetone was used as blank and baseline correction. Sample dilutions were made with acetone to avoid saturation of the spectrophotometric monitoring of the extracts (1:5 dilution for 0.5% and 1% starch extracts; 1:10 dilution for 0.5%, 1% and 1.5% glucose extracts; and 1:20 dilution for 1.5–2.5% starch and 2–2.5% glucose extracts). The spectra are represented with the dilution factor applied. 

### 3.3. In Vitro Antioxidant Activity

With the aim of determining the antioxidant activity of haloarchaeal carotenoid extracts, four assays were carried out based on previous research published by Loizzo and collaborators [89,90]. Acetone was removed, and serial dilutions were prepared with methanol. During the FRAP assay, the reduction of tripyridyltriazine (TPTZ)-Fe^3+^ by the carotenoids was quantified. The absorbance was read at 595 nm. Radical scavenging activity was assessed using 2,2-diphenyl-1-picrylhydrazyl (DPPH) and 2,2-azino-bis(3-ethylbenzothiazoline-6-sulfonic) acid (ABTS) assays. The DPPH assay is based on the spectrophotometric detection of the bleaching of the DPPH radical at 517 nm after 30 min of exposure to the samples. Regarding the ABTS assay, a mix containing the ABTS radical cation and the sample was prepared and incubated for 6 min in the dark. Then, the activity was established by measuring at 734 nm. The β-carotene bleaching test was carried out to evaluate the inhibition of lipid peroxidation by the samples.

### 3.4. Carbohydrate-Hydrolyzing Enzymes and Lipase Inhibitory Activities

The identification of compounds capable of inhibiting α-glucosidase and α-amylase is of much interest to the pharmaceutical and medical industries since these enzymes could be useful in the modulation of postprandial hyperglycemia; and therefore, in the management of the prediabetes condition. A pancreatic lipase inhibitory assay was used to determine the ability of the carotenoid extracts to reduce fat absorption. The protocols used were those described elsewhere by Loizzo and collaborators [82,89,90,91]. Samples were mixed with 4-nitrophenyl octanoate (NPC), Tris-HCl buffer (pH 8.5), and an enzyme solution in a 96-well plate and incubated at 37 °C for 30 min. The absorbance was measured at 405 nm.

### 3.5. Determination of the Composition of Carotenoid Extracts by HPLC Analysis

The HPLC analysis of carotenoids in acetone was performed using a Zorbax extended -C18 column (Agilent, Santa Clara, CA, USA) (2.1 × 50 mm, 1.8 µm) on an Agilent 1200 series system (Santa Clara, CA, USA). To determine the mass spectra of the different compounds, a 6490 Triple Quad LC/MS system (Agilent, Santa Clara, CA, USA) was used equipped with an electrospray ionization source (ESI) jet stream operating in positive scan mode (*m*/*z* range of 300–900) with 0.1 a.m.u (atomic mass unit) precision and controlled by MassHunter Workstation Software (Agilent, B.05.00, Santa Clara, CA, USA). The following specific working conditions were used: capillary voltage 3000 V, gas flow rate 11 L min^−1^, gas temperature 290 °C, sheath gas flow rate 12 L min^−1^, sheath gas temperature 300 °C, and nebulizer pressure 35 psi. The percentage represented by each carotenoid was calculated by dividing the sum of the areas of a carotenoid by the sum of the areas of all identified carotenoids.

### 3.6. Statistical Analysis

All experiments were carried out in triplicate. The results are expressed as the mean ± standard deviation (SD). Prism GraphPad Prism version 7.04 for Windows (GraphPad Software, San Diego, CA, USA) was used to determine the concentration of carotenoid extracts that exerted 50% inhibition (IC_50_). In the biological tests, differences within and between groups were evaluated by one-way ANOVA followed by a multicomparison Dunnett’s test. Pearson’s correlation coefficient (r), assessment of repeatability, linear regression, average, and relative standard deviation calculation were completed using Prism GraphPad Prism version 7.04 for Windows. The relative antioxidant capacity index (RACI) was calculated as described elsewhere [64,92]. All samples were ranked by the IC_50_ values and the SD. The dimensionless score was obtained by subtracting the mean from the raw data divided by the standard deviation. The standard scores of a sample for different antioxidant assays were calculated by combining data from different methods without unit limitation and no variance among techniques. All antioxidant assays were included, i.e., DPPH, ABTS, FRAP, and β-carotene bleaching tests. 

## Figures and Tables

**Table 1:** Radical scavenging activity and antioxidant capacity of BR methanolic extracts from *Hfx. mediterranei* R-4 under different cell culture conditions.

Sample	DPPH Test IC_50_ (µg/mL)	ABTS Test IC_50_ (µg/mL)	FRAP Test µM Fe (II)/g	β-Carotene Bleaching Test IC_50_ (µg/mL)	
				30 min	60 min
Control	120.0 ± 4.8	2.9 ± 0.1	18.1 ± 1.4	12.1 ± 1.4	28.1 ± 2.2
0.5% Glucose	73.8 ± 8.2 ****	0.23 ± 0.058 ****	20.0 ± 1.6 ns	14.1 ± 0.7 **	21.0 ± 2.1 ***
1% Glucose	40.3 ± 4.1 ****	0.12 ± 0.029 ****	25.3 ± 1.3 ****	1.5 ± 0.2 ****	11.9 ± 3.9 ****
1.5% Glucose	39.4 ± 4.3 ****	0.05 ± 0.004 ****	30.7 ± 1.1 ****	0.51 ± 0.07 ****	0.75 ± 0.09 ****
2% Glucose	35.2 ± 2.9 ****	0.04 ± 0.005 ****	38.7 ± 1.1 ****	0.49 ± 0.08 ****	0.65 ± 0.01 ****
2.5% Glucose	32.4 ± 3.6 ****	0.03 ± 0.003 ****	39.5 ± 0.7 ****	0.15 ± 0.01 ****	0.47 ± 0.04 ****
0.5% Starch	54.4 ± 5.1 ****	2.0 ± 0.5 ****	19.6 ± 1.4 ns	4.1 ± 0.8 ****	10.2 ± 0.1 ****
1% Starch	53.0 ± 5.3 ****	0.10 ± 0.012 ****	21.0 ± 1.1 *	2.0 ± 0.3 ****	4.3 ± 0.4 ****
1.5% Starch	51.4 ± 6.9 ****	0.10 ± 0.004 ****	22.3 ±0.8 ***	1.4 ± 0.2 ****	2.0 ± 0.2 ****
2% Starch	47.6 ± 5.6 ****	0.05 ± 0.003 ****	24.2 ± 0.9 ****	0.28 ± 0.06 ****	1.6 ± 0.08 ****
2.5% Starch	40.6 ± 5.0 ****	0.04 ± 0.006 ****	30.5 ± 1.4 ****	0.15 ± 0.008 ****	1.1 ± 0.11 ****

Data are given as means ± S.D. (*n* = 3); DPPH radical scavenging activity assay, antioxidant capacity determined by radical cation (ABTS^+^), β-carotene bleaching test, ferric ion reducing antioxidant power (FRAP), and relative antioxidant capacity index (RACI). Differences between groups were evaluated by one-way ANOVA followed by a multicomparison Dunnett’s test (α = 0.05) compared with the BR extract of the control cell culture (without glucose or starch). **** *p* < 0.0001, *** *p* < 0.001, ** *p* < 0.01, * *p* < 0.05, ns: not statistically significant. Positive controls: Ascorbic acid for DPPH and ABTS (IC_50_ values of 5.1 ± 0.8 and 1.7 ± 0.06 µg/mL, respectively); BHT for FRAP (63.1 ± 4.3 µM Fe (II)/g); propyl gallate for the β-carotene bleaching test at 30 and 60 min (IC_50_ values of 1.0 ± 0.04 and 0.09 ± 0.004 µg/mL, respectively).

**Table 2:** α-amylase, α-glucosidase, and lipase inhibitory activity (IC_50_ (µg/mL)) of BR methanolic extracts from *Hfx. mediterranei* R-4 under different cell culture conditions.

Sample	α-Glucosidase Test IC_50_ (µg/mL)	α-Amylase Test IC_50_ (µg/mL)	Lipase Test IC_50_ (µg/mL)
Control	117.2 ± 3.2	86.9 ± 8.3	78.4 ± 5.6
0.5% Glucose	73.1 ± 9.7 ****	23.7 ± 3.5 ****	80.5 ± 3.5 ns
1% Glucose	25.4 ± 4.6 ****	11.5 ± 1.3 ****	75.2 ± 5.9 ns
1.5% Glucose	11.9 ± 2.9 ****	5.1 ± 0.6 ****	68.3 ± 3.2 ns
2% Glucose	6.0 ± 1.3 ****	1.9 ± 0.07 ****	24.7 ± 0.8 ****
2.5% Glucose	3.2 ± 0.6 ****	1.3 ± 0.1 ****	5.3 ± 0.4 ****
0.5% Starch	79.8 ± 8.8 ****	18.8 ± 3.1 ****	86.0 ± 4.2 ns
1% Starch	21.0 ± 2.8 ****	12.7 ± 0.9 ****	77.8 ± 3.9 ns
1.5% Starch	13.8 ± 3.3 ****	3.4 ± 0.4 ****	70.5 ± 2.6 ns
2% Starch	7.3 ± 1.3 ****	1.8 ± 0.2 ****	46.5 ± 9.7 ****
2.5% Starch	3.6 ± 0.8 ****	1.4 ± 0.1 ****	5.3 ± 0.6 ****

Data are expressed as means ± S.D. (*n* = 3). Differences between groups were evaluated by one-way ANOVA followed by a multicomparison Dunnett’s test (*α* = 0.05) compared with the BR extract of the control cell culture (without glucose or starch). **** *p* < 0.0001. ns: not statistically significant. Positive controls: Acarbose for α-glucosidase and α-amylase tests (IC_50_ values of 35.6 ± 0.91 and 50.2 ± 1.3 µg/mL, respectively) and Orlistat for the lipase test (IC_50_ values of 37.5 ± 1.0 µg/mL).

**Table 3:** Previously reported retention times for each BR isomer and BR retention times detected in *Hfx. mediterranei* carotenoid extracts (present study).

BR Isomer	Retention Times (min)		
	Flores et al., 2020	Lizama et al., 2021	*Hfx. mediterranei* (Present Results)
All *trans*-BR	10.4	4.45	3–4
5-*cis* BR	11.2	4.92	4.2–4.6
9-*cis* BR	11.7	5.91	4.8–5.2
13-*cis* BR	12.1	6.31	5.4–5.7
5-*cis*-26-*cis*-BR	-	7.02	7–7.9
9-*cis*-26-*cis*-BR	-	8.04	7–7.9

## References

[1] Rao A., Rao L. (2007). Carotenoids and Human Health. Pharmacol. Res..

[2] Rodriguez-Concepcion M., Avalos J., Bonet M.L., Boronat A., Gomez-Gomez L., Hornero-Mendez D., Limon M.C., Meléndez-Martínez A.J., Olmedilla-Alonso B., Palou A. (2018). A Global Perspective on Carotenoids: Metabolism, Biotechnology, and Benefits for Nutrition and Health. Prog. Lipid Res..

[3] Milani A., Basirnejad M., Shahbazi S., Bolhassani A. (2017). Carotenoids: Biochemistry, Pharmacology and Treatment. Br. J. Pharmacol..

[4] Giani M., Montoyo-Pujol Y.G., Peiró G., Martínez-Espinosa R.M. (2021). Halophilic Carotenoids and Breast Cancer: From Salt Marshes to Biomedicine. Mar. Drugs.

[5] Yabuzaki J. (2017). Carotenoids Database: Structures, Chemical Fingerprints and Distribution among Organisms. Database.

[6] Giani M., Garbayo I., Vílchez C., Martínez-Espinosa R.M. (2019). Haloarchaeal Carotenoids: Healthy Novel Compounds from Extreme Environments. Mar. Drugs.

[7] Oren A. (2020). The Microbiology of Red Brines. Advances in Applied Microbiology.

[8] Oren A. (2015). Halophilic Microbial Communities and Their Environments. Curr. Opin. Biotechnol..

[9] Ventosa A., Oren A., Ma Y. (2011). Halophiles and Hypersaline Environments: Current Research and Future Trends.

[10] Jones D.L., Baxter B.K. (2017). DNA Repair and Photoprotection: Mechanisms of Overcoming Environmental Ultraviolet Radiation Exposure in Halophilic Archaea. Front. Microbiol..

[11] Kelly M., Jensen S.L., Theander O., Cyvin S.J., Hagen G. (1967). Bacterial Carotenoids. XXVI. C50-Carotenoids. 2. Bacterioruberin. Acta Chem. Scand..

[12] Bidle K.A., Hanson T.E., Howell K., Nannen J. (2007). HMG-CoA Reductase Is Regulated by Salinity at the Level of Transcription in *Haloferax volcanii*. Extremophiles.

[13] Torregrosa-Crespo J., Galiana C.P., Martínez-Espinosa R.M., Sghaier H., Najjari A., Ghedira K. (2017). Biocompounds from Haloarchaea and Their Uses in Biotechnology. Archaea—New Biocatalysts, Novel Pharmaceuticals and Various Biotechnological Applications.

[14] Saito T., Miyabe Y., Ide H., Yamamoto O. (1997). Hydroxyl Radical Scavenging Ability of Bacterioruberin. Radiat. Phys. Chem..

[15] Yatsunami R., Ando A., Yang Y., Takaichi S., Kohno M., Matsumura Y., Ikeda H., Fukui T., Nakasone K., Fujita N. (2014). Identification of Carotenoids from the Extremely Halophilic Archaeon *Haloarcula japonica*. Front. Microbiol..

[16] Rodrigo-Baños M., Garbayo I., Vílchez C., Bonete M., Martínez-Espinosa R. (2015). Carotenoids from Haloarchaea and Their Potential in Biotechnology. Mar. Drugs.

[17] Hegazy G.E., Abu-Serie M.M., Abo-Elela G.M., Ghozlan H., Sabry S.A., Soliman N.A., Abdel-Fattah Y.R. (2020). In Vitro Dual (Anticancer and Antiviral) Activity of the Carotenoids Produced by Haloalkaliphilic Archaeon *Natrialba* sp. M6. Sci. Rep..

[18] Zalazar L., Pagola P., Miró M.V., Churio M.S., Cerletti M., Martínez C., Iniesta-Cuerda M., Soler A.J., Cesari A., de Castro R. (2019). Bacterioruberin Extracts from a Genetically Modified Hyperpigmented *Haloferax volcanii* Strain: Antioxidant Activity and Bioactive Properties on Sperm Cells. J. Appl. Microbiol..

[19] Flores N., Hoyos S., Venegas M., Galetović A., Zúñiga L.M., Fábrega F., Paredes B., Salazar-Ardiles C., Vilo C., Ascaso C. (2020). *Haloterrigena* Sp. Strain SGH1, a Bacterioruberin-Rich, Perchlorate-Tolerant Halophilic Archaeon Isolated From Halite Microbial Communities, Atacama Desert, Chile. Front. Microbiol..

[20] Sahli K., Gomri M.A., Esclapez J., Gómez-Villegas P., Bonete M.-J., León R., Kharroub K. (2022). Characterization and Biological Activities of Carotenoids Produced by Three Haloarchaeal Strains Isolated from Algerian Salt Lakes. Arch. Microbiol..

[21] Henke N.A., Frohwitter J., Peters-Wendisch P., Wendisch V.F., Barreiro C., Barredo J.-L. (2018). Carotenoid Production by Recombinant Corynebacterium Glutamicum: Strain Construction, Cultivation, Extraction, and Quantification of Carotenoids and Terpenes. Microbial Carotenoids.

[22] Mussagy C.U., Winterburn J., Santos-Ebinuma V.C., Pereira J.F.B. (2019). Production and Extraction of Carotenoids Produced by Microorganisms. Appl. Microbiol. Biotechnol..

[23] Mata-Gómez L.C., Montañez J.C., Méndez-Zavala A., Aguilar C.N. (2014). Biotechnological Production of Carotenoids by Yeasts: An Overview. Microb. Cell Fact..

[24] Montero-Lobato Z., Ramos-Merchante A., Fuentes J., Sayago A., Fernández-Recamales Á., Martínez-Espinosa R., Vega J., Vílchez C., Garbayo I. (2018). Optimization of Growth and Carotenoid Production by *Haloferax mediterranei* Using Response Surface Methodology. Mar. Drugs.

[25] Naziri D., Hamidi M., Hassanzadeh S., Tarhriz V., Maleki Zanjani B., Nazemyieh H., Hejazi M.A., Hejazi M.S. (2014). Analysis of Carotenoid Production by *Halorubrum* sp. TBZ126; an Extremely Halophilic Archeon from Urmia Lake. Adv. Pharm. Bull..

[26] Huang T.-Y., Duan K.-J., Huang S.-Y., Chen C.W. (2006). Production of Polyhydroxyalkanoates from Inexpensive Extruded Rice Bran and Starch by *Haloferax mediterranei*. J. Ind. Microbiol. Biotechnol..

[27] Quillaguamán J., Guzmán H., Van-Thuoc D., Hatti-Kaul R. (2010). Synthesis and Production of Polyhydroxyalkanoates by Halophiles: Current Potential and Future Prospects. Appl. Microbiol. Biotechnol..

[28] Torregrosa-Crespo J., Martínez-Espinosa R.M., Esclapez J., Bautista V., Pire C., Camacho M., Richardson D.J., Bonete M.J. (2016). Anaerobic Metabolism in *Haloferax* Genus. Advances in Microbial Physiology.

[29] Giani M., Montero-Lobato Z., Garbayo I., Vílchez C., Vega J.M., Martínez-Espinosa R.M. (2021). *Haloferax mediterranei* Cells as C_50_ Carotenoid Factories. Mar. Drugs.

[30] Koller M., Maršálek L., de Sousa Dias M.M., Braunegg G. (2017). Producing Microbial Polyhydroxyalkanoate (PHA) Biopolyesters in a Sustainable Manner. New Biotechnol..

[31] Wang K., Zhang R. (2021). Production of Polyhydroxyalkanoates (PHA) by *Haloferax mediterranei* from Food Waste Derived Nutrients for Biodegradable Plastic Applications. J. Microbiol. Biotechnol..

[32] Simó-Cabrera L., García-Chumillas S., Hagagy N., Saddiq A., Tag H., Selim S., AbdElgawad H., Arribas Agüero A., Monzó Sánchez F., Cánovas V. (2021). Haloarchaea as Cell Factories to Produce Bioplastics. Mar. Drugs.

[33] Cánovas V., Garcia-Chumillas S., Monzó F., Simó-Cabrera L., Fernández-Ayuso C., Pire C., Martínez-Espinosa R.M. (2021). Analysis of Polyhydroxyalkanoates Granules in *Haloferax mediterranei* by Double-Fluorescence Staining with Nile Red and SYBR Green by Confocal Fluorescence Microscopy. Polymers.

[34] Torregrosa-Crespo J., Pire C., Bergaust L., Martínez-Espinosa R.M. (2020). *Haloferax mediterranei*, an Archaeal Model for Denitrification in Saline Systems, Characterized Through Integrated Physiological and Transcriptional Analyses. Front. Microbiol..

[35] Hou J., Cui H.-L. (2018). In Vitro Antioxidant, Antihemolytic, and Anticancer Activity of the Carotenoids from Halophilic Archaea. Curr. Microbiol..

[36] Squillaci G., Parrella R., Carbone V., Minasi P., La Cara F., Morana A. (2017). Carotenoids from the Extreme Halophilic Archaeon *Haloterrigena turkmenica*: Identification and Antioxidant Activity. Extremophiles.

[37] D’Souza S.E., Altekar W., D’Souza S.F. (1997). Adaptive Response of Haloferax mediterranei to Low Concentrations of NaCl (<20%) in the Growth Medium. Arch. Microbiol..

[38] Glovaci D., Fan W., Wong N.D. (2019). Epidemiology of Diabetes Mellitus and Cardiovascular Disease. Curr. Cardiol. Rep..

[39] Huang H., Yan Z., Chen Y., Liu F. (2016). A Social Contagious Model of the Obesity Epidemic. Sci. Rep..

[40] Jaacks L.M., Vandevijvere S., Pan A., McGowan C.J., Wallace C., Imamura F., Mozaffarian D., Swinburn B., Ezzati M. (2019). The Obesity Transition: Stages of the Global Epidemic. Lancet Diabetes Endocrinol..

[41] Ortega F.B., Lavie C.J., Blair S.N. (2016). Obesity and Cardiovascular Disease. Circ. Res..

[42] Balakumar P., Maung-U K., Jagadeesh G. (2016). Prevalence and Prevention of Cardiovascular Disease and Diabetes Mellitus. Pharmacol. Res..

[43] Baron A.D. (1998). Postprandial Hyperglycaemia and α-Glucosidase Inhibitors. Diabetes Res. Clin. Pract..

[44] Chiasson J.-L., Josse R.G., Gomis R., Hanefeld M., Karasik A., Laakso M. (2002). Acarbose for Prevention of Type 2 Diabetes Mellitus: The STOP-NIDDM Randomised Trial. Lancet.

[45] Hu R., Li Y., Lv Q., Wu T., Tong N. (2015). Acarbose Monotherapy and Type 2 Diabetes Prevention in Eastern and Western Prediabetes: An Ethnicity-Specific Meta-Analysis. Clin. Ther..

[46] Kumar A., Chauhan S. (2021). Pancreatic Lipase Inhibitors: The Road Voyaged and Successes. Life Sci..

[47] Bialecka-Florjanczyk E., Fabiszewska A.U., Krzyczkowska J., Kurylowicz A. (2018). Synthetic and Natural Lipase Inhibitors. Mini Rev. Med. Chem..

[48] Kuprat T., Ortjohann M., Johnsen U., Schönheit P. (2021). Glucose Metabolism and Acetate Switch in Archaea: The Enzymes in *Haloferax volcanii*. J. Bacteriol..

[49] Vázquez-Madrigal A.S., Barbachano-Torres A., Arellano-Plaza M., Kirchmayr M.R., Finore I., Poli A., Nicolaus B., de la Torre Zavala S., Camacho-Ruiz R.M. (2021). Effect of Carbon Sources in Carotenoid Production from *Haloarcula* sp. M1, *Halolamina* sp. M3 and *Halorubrum* sp. M5, Halophilic Archaea Isolated from Sonora Saltern, Mexico. Microorganisms.

[50] Will Chen C., Hsu S., Lin M.-T., Hsu Y. (2015). Mass Production of C_50_ Carotenoids by *Haloferax mediterranei* in Using Extruded Rice Bran and Starch under Optimal Conductivity of Brined Medium. Bioprocess Biosyst. Eng..

[51] Gochnauer M.B., Kushwaha S.C., Kates M., Kushner D.J. (1972). Nutritional Control of Pigment and Isoprenoid Compound Formation in Extremely Halophilic Bacteria. Archiv. Mikrobiol..

[52] Mandelli F., Miranda V.S., Rodrigues E., Mercadante A.Z. (2012). Identification of Carotenoids with High Antioxidant Capacity Produced by Extremophile Microorganisms. World J. Microbiol. Biotechnol..

[53] Melendez-Martínez A., Britton G., Vicario I., Heredia F. (2006). HPLC Analysis of Geometrical Isomers of Lutein Epoxide Isolated from Dandelion (*Taraxacum officinale* F. Weber Ex Wiggers). Phytochemistry.

[54] Cerletti M., Paggi R., Troetschel C., Ferrari M.C., Guevara C.R., Albaum S., Poetsch A., de Castro R. (2018). LonB Protease Is a Novel Regulator of Carotenogenesis Controlling Degradation of Phytoene Synthase in *Haloferax volcanii*. J. Proteome Res..

[55] Abbes M., Baati H., Guermazi S., Messina C., Santulli A., Gharsallah N., Ammar E. (2013). Biological Properties of Carotenoids Extracted from *Halobacterium halobium* Isolated from a Tunisian Solar Saltern. BMC Complement. Altern. Med..

[56] Kumar P., Jun H.-B., Kim B.S. (2018). Co-Production of Polyhydroxyalkanoates and Carotenoids through Bioconversion of Glycerol by *Paracoccus* sp. Strain LL1. Int. J. Biol. Macromol..

[57] Gómez-Villegas P., Vigara J., Vila M., Varela J., Barreira L., Léon R. (2020). Antioxidant, Antimicrobial, and Bioactive Potential of Two New Haloarchaeal Strains Isolated from Odiel Salterns (Southwest Spain). Biology.

[58] Lizama C., Romero-Parra J., Andrade D., Riveros F., Bórquez J., Ahmed S., Venegas-Salas L., Cabalín C., Simirgiotis M.J. (2021). Analysis of Carotenoids in Haloarchaea Species from Atacama Saline Lakes by High Resolution UHPLC-Q-Orbitrap-Mass Spectrometry: Antioxidant Potential and Biological Effect on Cell Viability. Antioxidants.

[59] Sahli K., Gomri M.A., Esclapez J., Gómez-Villegas P., Ghennai O., Bonete M.-J., León R., Kharroub K. (2020). Bioprospecting and Characterization of Pigmented Halophilic Archaeal Strains from Algerian Hypersaline Environments with Analysis of Carotenoids Produced by *Halorubrum* sp. BS2. J. Basic Microbiol..

[60] Dose J., Matsugo S., Yokokawa H., Koshida Y., Okazaki S., Seidel U., Eggersdorfer M., Rimbach G., Esatbeyoglu T. (2016). Free Radical Scavenging and Cellular Antioxidant Properties of Astaxanthin. Int. J. Mol. Sci..

[61] Hu C.-C., Lin J.-T., Lu F.-J., Chou F.-P., Yang D.-J. (2008). Determination of Carotenoids in *Dunaliella salina* Cultivated in Taiwan and Antioxidant Capacity of the Algal Carotenoid Extract. Food Chem..

[62] Singh P., Baranwal M., Reddy S.M. (2016). Antioxidant and Cytotoxic Activity of Carotenes Produced by *Dunaliella salina* under Stress. Pharm. Biol..

[63] Bellahcen T.O., AAmiri A., Touam I., Hmimid F., Amrani A.E., Cherif A., Cherki M. (2020). Evaluation of Moroccan Microalgae: *Spirulina platensis* as a Potential Source of Natural Antioxidants. J. Complement. Integr. Med..

[64] Sun T., Tanumihardjo S.A. (2007). An Integrated Approach to Evaluate Food Antioxidant Capacity. J Food Sci..

[65] Rani V., Deep G., Singh R.K., Palle K., Yadav U.C.S. (2016). Oxidative Stress and Metabolic Disorders: Pathogenesis and Therapeutic Strategies. Life Sci..

[66] Karam B.S., Chavez-Moreno A., Koh W., Akar J.G., Akar F.G. (2017). Oxidative Stress and Inflammation as Central Mediators of Atrial Fibrillation in Obesity and Diabetes. Cardiovasc. Diabetol..

[67] Phan M.A.T., Wang J., Tang J., Lee Y.Z., Ng K. (2013). Evaluation of α-Glucosidase Inhibition Potential of Some Flavonoids from Epimedium Brevicornum. LWT-Food Sci. Technol..

[68] Zhang L., Tu Z., Yuan T., Wang H., Xie X., Fu Z. (2016). Antioxidants and α-Glucosidase Inhibitors from *Ipomoea batatas* Leaves Identified by Bioassay-Guided Approach and Structure-Activity Relationships. Food Chem..

[69] Chen G., Guo M. (2017). Rapid Screening for α-Glucosidase Inhibitors from *Gymnema sylvestre* by Affinity Ultrafiltration–HPLC-MS. Front. Pharmacol..

[70] Hong H.-C., Li S.-L., Zhang X.-Q., Ye W.-C., Zhang Q.-W. (2013). Flavonoids with α-Glucosidase Inhibitory Activities and Their Contents in the Leaves of *Morus atropurpurea*. Chin. Med..

[71] Hou W., Li Y., Zhang Q., Wei X., Peng A., Chen L., Wei Y. (2009). Triterpene Acids Isolated from *Lagerstroemia speciosa* Leaves as *α*-Glucosidase Inhibitors: Triterpene acids as alpha-glucosidase inhibitors. Phytother. Res..

[72] Abbas G., Al Harrasi A., Hussain H., Hamaed A., Supuran C.T. (2019). The Management of Diabetes Mellitus-Imperative Role of Natural Products against Dipeptidyl Peptidase-4, α-Glucosidase and Sodium-Dependent Glucose Co-Transporter 2 (SGLT2). Bioorg. Chem..

[73] Ramírez G., Zavala M., Pérez J., Zamilpa A. (2012). In Vitro Screening of Medicinal Plants Used in Mexico as Antidiabetics with Glucosidase and Lipase Inhibitory Activities. Evid.-Based Complement. Altern. Med..

[74] Li J., Chi G., Wang L., Wang F., He S. (2020). Isolation, Identification, and Inhibitory Enzyme Activity of Phenolic Substances Present in *Spirulina*. J. Food Biochem..

[75] Prabakaran G., Sampathkumar P., Kavisri M., Moovendhan M. (2020). Extraction and Characterization of Phycocyanin from *Spirulina platensis* and Evaluation of Its Anticancer, Antidiabetic and Antiinflammatory Effect. Int. J. Biol. Macromol..

[76] Hwang P.-A., Hung Y.-L., Tsai Y.-K., Chien S.-Y., Kong Z.-L. (2015). The Brown Seaweed *Sargassum hemiphyllum* Exhibits α-Amylase and α-Glucosidase Inhibitory Activity and Enhances Insulin Release in Vitro. Cytotechnology.

[77] Unnikrishnan P., Suthindhiran K., Jayasri M. (2015). Alpha-Amylase Inhibition and Antioxidant Activity of Marine Green Algae and Its Possible Role in Diabetes Management. Pharmacogn. Mag..

[78] Lowe M.E. (1997). Structure and Function of Pancreatic Lipase and Colipase. Annu. Rev. Nutr..

[79] Zhang R., Xing D., Wang C. (2021). Pancreatic Triglyceride Lipase Inhibitors Derived from Natural Products: How to Dig into the Truth. J. Agric. Food Chem..

[80] Samuel Wu Y.-H., Chiu C.-H., Yang D.-J., Lin Y.-L., Tseng J.-K., Chen Y.-C. (2013). Inhibitory Effects of Litchi (*Litchi chinensis* Sonn.) Flower-Water Extracts on Lipase Activity and Diet-Induced Obesity. J. Funct. Foods.

[81] Matsuo Y., Matsumoto K., Inaba N., Mimaki Y. (2018). Daisaikoto Inhibits Pancreatic Lipase Activity and Decreases Serum Triglyceride Levels in Mice. Biol. Pharm. Bull..

[82] Loizzo M.R., Marrelli M., Pugliese A., Conforti F., Nadjafi F., Menichini F., Tundis R. (2016). *Crocus cancellatus* subsp. Damascenus Stigmas: Chemical Profile, and Inhibition of α -Amylase, α-Glucosidase and Lipase, Key Enzymes Related to Type 2 Diabetes and Obesity. J. Enzym. Inhib. Med. Chem..

[83] Ronnekleiv M. (1995). Bacterial Carotenoids 53∗ C_50_-Carotenoids 23; Carotenoids of *Haloferax volcanii* versus Other Halophilic Bacteria. Biochem. Syst. Ecol..

[84] Fang C.-J., Ku K.-L., Lee M.-H., Su N.-W. (2010). Influence of Nutritive Factors on C_50_ Carotenoids Production by *Haloferax mediterranei* ATCC 33500 with Two-Stage Cultivation. Bioresour. Technol..

[85] Asker D., Awad T., Ohta Y. (2002). Lipids of *Haloferax alexandrinus* strain TMT: An Extremely Halophilic Canthaxanthin-Producing Archaeon. J. Biosci. Bioeng..

[86] De la Vega M., Sayago A., Ariza J., Barneto A.G., León R. (2016). Characterization of a Bacterioruberin-Producing Haloarchaea Isolated from the Marshlands of the Odiel River in the Southwest of Spain. Biotechnol. Prog..

[87] Asker D., Ohta Y. (1999). Production of Canthaxanthin by Extremely Halophilic Bacteria. J. Biosci. Bioeng..

[88] Rodriguez-Valera F., Ruiz-Berraquero F., Ramos-Cormenzana A. (1980). Behaviour of Mixed Populations of Halophilic Bacteria in Continuous Cultures. Can. J. Microbiol..

[89] Loizzo M.R., Leporini M., Sicari V., Falco T., Pellicanò T.M., Tundis R. (2018). Investigating the in Vitro Hypoglycaemic and Antioxidant Properties of *Citrus* × *Clementina* Hort. Juice. Eur. Food Res. Technol..

[90] Loizzo M.R., Pugliese A., Bonesi M., de Luca D., O’Brien N., Menichini F., Tundis R. (2013). Influence of Drying and Cooking Process on the Phytochemical Content, Antioxidant and Hypoglycaemic Properties of Two Bell *Capsicum annum* L. Cultivars. Food Chem. Toxicol..

[91] Loizzo M., Lucci P., Núñez O., Tundis R., Balzano M., Frega N., Conte L., Moret S., Filatova D., Moyano E. (2019). Native Colombian Fruits and Their By-Products: Phenolic Profile, Antioxidant Activity and Hypoglycaemic Potential. Foods.

[92] Gorjanović S.Ž., Alvarez-Suarez J.M., Novaković M.M., Pastor F.T., Pezo L., Battino M., Sužnjević D.Ž. (2013). Comparative Analysis of Antioxidant Activity of Honey of Different Floral Sources Using Recently Developed Polarographic and Various Spectrophotometric Assays. J. Food Compos. Anal..

